# Genetic variants predicting left ventricular hypertrophy in a diabetic population: a Go-DARTS study including meta-analysis

**DOI:** 10.1186/1475-2840-12-109

**Published:** 2013-07-23

**Authors:** Helen M Parry, Louise A Donnelly, Natalie Van Zuydam, Alexander SF Doney, Douglas HJ Elder, Andrew D Morris, Alan D Struthers, Colin NA Palmer, Chim C Lang

**Affiliations:** 1 Division of Cardiovascular and Diabetes Medicine, Level 7, Mailbox 2,Ninewells Hospital and Medical School, University of Dundee, Dundee DD19SY, UK; 2 Pat McPherson Centre for Pharmacogenomics and Pharmacogenetics, Division of Cardiovascular and Diabetes Medicine, Ninewells Hospital and Medical School, University of Dundee, Dundee, UK

**Keywords:** Left ventricular hypertrophy, Genetics, Type 2 diabetes mellitus

## Abstract

**Background:**

Left ventricular hypertrophy has multiple aetiologies including diabetes and genetic factors. We aimed to identify genetic variants predicting left ventricular hypertrophy in diabetic individuals.

**Methods:**

Demographic, echocardiographic, prescribing, morbidity, mortality and genotyping databases connected with the Genetics of Diabetes Audit and Research in Tayside, Scotland project were accurately linked using a patient-specific identifier. Left ventricular hypertrophy cases were identified using echocardiographic data.

Genotyping data from 973 cases and 1443 non-left ventricular hypertrophy controls were analysed, investigating whether single nucleotide polymorphisms associated with left ventricular hypertrophy in previous Genome Wide Association Studies predicted left ventricular hypertrophy in our population of individuals with type 2 diabetes. Meta-analysis assessed overall significance of these single nucleotide polymorphisms, which were also used to create gene scores. Logistic regression assessed whether these scores predicted left ventricular hypertrophy.

**Results:**

Two single nucleotide polymorphisms previously associated with left ventricular hypertrophy were significant: rs17132261: OR 2.03, 95% CI 1.10-3.73, p-value 0.02 and rs2292462: OR 0.82, 95% CI 0.73-0.93 and p-value 2.26x10^-3^. Meta-analysis confirmed rs17132261 and rs2292462 were associated with left ventricular hypertrophy (p=1.03x10^-8^ and p=5.86x10^-10^ respectively) and one single nucleotide polymorphisms in IGF1R (rs4966014) became genome wide significant upon meta-analysis although was not significant in our study. Gene scoring based on published single nucleotide polymorphisms also predicted left ventricular hypertrophy in our study.

Rs17132261, within SLC25A46, encodes a mitochondrial phosphate transporter, implying abnormal myocardial energetics contribute to left ventricular hypertrophy development. Rs2292462 lies within the obesity-implicated neuromedin B gene. Rs4966014 lies within the IGF1R1 gene. IGF1 signalling is an established factor in cardiac hypertrophy.

**Conclusions:**

We created a resource to study genetics of left ventricular hypertrophy in diabetes and validated our left ventricular hypertrophy phenotype in replicating single nucleotide polymorphisms identified by previous genome wide association studies investigating left ventricular hypertrophy.

## Background

Cardiovascular complications are the most important cause of mortality and morbidity in patients with type 2 diabetes mellitus (T2DM), accounting for around two thirds of total mortality [1]. Increased cardiovascular risk may be partly related to a direct adverse effect on the heart in T2DM, independent of coronary artery disease [2]. T2DM can directly affect the myocardium, promoting left ventricular hypertrophy (LVH) [3].

LVH strongly predicts adverse cardiovascular sequelae including overall cardiovascular mortality, myocardial infarction, development of heart failure and cerebrovascular events [4-7]. LVH is common in T2DM and T2DM is an independent risk factor for LVH [8,9]. A large proportion of patients with T2DM and no overt cardiovascular disease have LVH [9]. Hypertension and obesity often co-exist with T2DM, contributing to LVH development. However, evidence shows LVH is independently associated with T2DM when covariates such as age, body mass index and hypertension are accounted for [2,10,11]. Not all patients with T2DM develop LVH, and those developing LVH do so to varying degrees [11]. This heterogeneity is likely to have a partial genetic basis. Indeed, LV mass heritability in non-diabetic subjects has been estimated through twin studies [12], studies in hypertensive siblings [13] and through complex family studies [14,15]. Maternally transmitted genetic susceptibility to LVH in T2DM has also been reported [16].

These studies have prompted investigation of the genetic basis of LVH through candidate gene studies and genome wide association studies (GWAS). Early studies seeking association between candidate genes and LVH looked at exclusively hypertensive cohorts [17,18], whilst other studies looked at candidate genes by comparing monozygotic and dizygotic twins [19].

Two population-based GWAS have sought single nucleotide polymorphisms (SNPs) associated with LVH in the general population [20,21]. Vasan *et al.*, using data collected by the EchoGen consortium (n=12612), found 2 SNPs associated with increased LV mass and 3 SNPs associated with increased LV wall thickness at genome wide significance. These SNPs were not replicated in the independent cohort [20]. Shah and colleagues looked at 3 population-based cohorts: The British Women's Heart and Health Study (BWHHS, n=3443), the Genetic Regulation of Arterial Pressure of Humans In the Community (GRAPHIC) Study (n=2024) and the Whitehall II Study (WHII) (n=5059). They found 4 SNPs associated with LVH defined by ECG criteria with genome wide significance and replicated in an independent cohort [21]. No studies to date have identified genes predicting development of LVH specifically in patients with T2DM.

Our LVH population is comprised of diabetic individuals in Tayside participating in Go-DARTS, who underwent echocardiography for varying reasons. We aimed to identify individuals with echocardiographically defined LVH and demonstrate its clinical impact. We sought to replicate previous findings in non-diabetics in a large population of patients with T2DM.

## Methods

### Go-DARTS population

The Diabetes Audit and Research Tayside, Scotland (DARTS) project was based on linking clinical records by a patient-specific identifier, the Community Health Index (CHI) number, allowing the creation and maintenance of sophisticated regional health informatics systems by the Health Informatics Centre (HIC), University of Dundee. The on-going DARTS project electronically followed all living residents in Tayside, Scotland from 1 January 1996 (n=391 274 including 7 596 individuals with diabetes) through linking the clinical datasets described below with a high degree of reliability and accuracy. This approach was well-validated in previous publications [22].

Data used for this comprehensive record linkage were assimilated from multiple sources including the Community Health Index, containing demographic data, the Scottish Morbidity Records providing International Classification of Disease coding for hospital admissions from 1980 until present, the General Registrars’ Office, providing mortality data since 1998, the regional biochemistry database, containing all biochemistry assays performed from 1981 and data from all prescriptions dispensed in Tayside since 1989 were also used.

These datasets were linked to genotyping data through Go-DARTS, a genetic sub-study of DARTS comprised of a cohort of Caucasian individuals with T2DM attending diabetes clinics in Tayside [23]. Collection and analysis of data in DARTS and Go-DARTS was approved by the East of Scotland Research and Ethics Committee, in compliance with the declaration of Helsinki. All participants had given written consent for their data to be linked and analysed for research purposes.

The Go-DARTS population has been extensively described in previous studies [24,25]. Both the DARTS and Go-DARTS studies received ethical approval from the local boards. We studied 3641 individuals with T2DM recruited to Go-DARTS between December 1998 and January 2008.

### The Tayside echocardiography database

The Tayside echocardiography database, maintained by the Department of Cardiology, contains data from all clinically requested echocardiograms performed in Ninewells Hospital, Dundee between 1994 and 2011. Echocardiograms were reported by British Society of Echocardiography (BSE) accredited echocardiographers. This database was used to identify echocardiographically defined cases of LVH and linked to the above datasets. The echocardiography database was previously linked to the health informatics system at HIC to identify structural heart disease [26,27]. Written permission was obtained from the echodatabase Caldicott guardian for this data linkage.

### LVH phenotype definition

LV measurements were used to identify individuals with LVH according to ASE criteria [28,29]. LV measurements were used to calculate LV mass according to the formula:

$$\text{LVmass}=0.8*\left(1.04*\left(\left(\text{LVIDD}+\text{LVPW}+\text{IVS}\right)ˆ\left(3\right)‒\left(\text{LVIDD}\right)ˆ\left(3\right)\right)\right)+0.6$$

derived by Devereux *et al.*[29,30], where LVIDD represented left ventricular internal diameter in diastole, LVPW referred to left ventricular posterior wall thickness and IVS was septal thickness. LV mass was separately indexed to height in metres to the power 2.7 and to body surface area (BSA) using the formula [31]:

$$\text{BSA}=\left({\left(\text{weight}\right)}^{0.425}\times {\left(\text{height}\right)}^{0.725}\right)*0.007184$$

Individuals were classed as having LVH if their LV mass was outside the normal range when indexed to either height or BSA using ASE cut-off values [28]. Participants with LV wall thickness exceeding the normal range according to direct 2D measures were also classed as cases of LVH. Additionally, individuals were regarded as having LVH if relative wall thickness (RWT) was increased using the formula [32]:

$$\text{RWT}=\left(2x\;\text{LVPW}\right)/\text{LVIDD}$$

Hence, inclusion criteria for LVH cases were:

1) Diagnosis of type 2 diabetes mellitus

2) LVH on echocardiography according to any of the above definitions using ASE cut-off values [28].

3) Absence of aortic stenosis greater than mild severity. As aortic stenosis promotes LVH through increased haemodynamic load on the left ventricle, these patients were excluded from further analysis to prevent confounding.

Genetic samples from individuals meeting all 3 inclusion criteria were taken forward for further analysis.

### Controls

Patients included in the analysis as control subjects met with the following criteria:

1) Diagnosis of type 2 diabetes mellitus

2) No clinically requested echocardiogram

3) Never received a prescription for a loop diuretic. Patients who had been prescribed a loop diuretic at any point were identified by extracting those with prescriptions issued for BNF code 2.2.2.

Genetic samples from individuals meeting all 3 inclusion criteria were taken forward for further analysis as non-LVH controls, comparing them to LVH cases as defined above.

### Glycaemic control

Mean glycosylated haemoglobin levels (HbA1C) were calculated for cases and controls and compared.

### Genotyping

Samples were genotyped at the Affymetrix service laboratory on the Genome-Wide Human SNP Array 6.0. Genotype quality control was via the standard protocol established for the Wellcome Trust Case Control Consortium 2 Study.

### Genotyping QC and sample QC

High density genotyping data were available for Go-DARTS participants. Four thousand individuals with T2DM typed on the Affymetrix 6.0 SNP genotyping array and genotypes were imputed into this population from a reference panel of HapMap2 individuals and a panel of 6000 British individuals typed on the Illumina dual using IMPUTE2 [33,34].

Genotype data quality control of the discovery samples was previously described [25,34,35].

### Analysis and statistical approaches

Data were analysed using SAS 9.2 for Windows and the R software [36]. Logistic regression analysis identified covariates influencing case–control status using values for clinical parameters (weight, height and blood pressure) taken closest to the echocardiogram date in cases of LVH and closest to the genotyping date in controls. Covariates shown to be significant were accounted for when genetic analysis was performed (see below). Survival analysis was used to show how LVH defined according to the above phenotype influenced morbidity and mortality to allow comparison with previous work.

### Replication previously identified SNPs associated with LVH

Logistic and linear regression modelling were performed assuming an additive genetic model using the PLINK v1.07 software (http://pngu.mgh.harvard.edu/~purcell/plink/[37]) and imputed data were modelled using the SCORE TEST in SNPTEST (https://mathgen.stats.ox.ac.uk/genetics_software/snptest/snptest.html[38]) that took genotype uncertainty into account. This allowed comparison of genetic variation at loci previously associated with LVH between cases of LVH and controls. P values less than 0.05 were taken as evidence of replication. Chi-square tests of independence were used to test the significance of replicated SNPs according to the above analysis. Cases were then divided into those with increased LV mass and those with concentric/eccentric remodelling and logistic regression modelling was repeated.

Survival analysis was performed to determine whether variations in these replicated SNPs influenced the likelihood of death and hospitalisation due to cardiovascular illness.

### Meta-analysis

Meta-analysis was performed to assess the likelihood the above SNPs were associated with LVH when our data was combined with published data. The odds ratios calculated for the published SNPs were based on continuous data so our binary case–control model was adjusted to allow comparison with published data. Continuous data for LV thickness and LV mass were used as outcome measures. Individual LV mass values were based on calculations using the equation detailed above and values for LV thickness were taken as the sum of the IVS and LVPW. A linear regression model was used to determine whether published SNPs associated with LV mass were associated with continuous measures of LV mass in this study. Further linear regression modelling was undertaken to assess whether either of the published SNPs associated specifically with LV thickness were associated with continuous measures of LV thickness in this study. These results were then meta-analysed using the weighted z values calculated using the inverse variance-weighted z-score as described by Bakker *et al.*[39] since the units used in the published GWAS differed from our own.

### Gene score calculation

SNPs discovered in GWAS meta-analyses [20,21] looking at LVH were combined into a gene score by rating the LVH raising allele by the meta-analysis effect using the ‘score’ option in PLINK [37].

SNPs discovered by Vasan *et al.*[20] were combined to produce gene score 1, which included rs17568359, rs7565161, rs7910620, rs2059238 and rs17132261. SNPs discovered by Shah *et al.*[21] were combined to produce gene score 2, including rs6797133, rs2292462, rs2290893 and rs4966014. All the above SNPs discovered by both Vasan *et al.* and Shah *et al.* were combined to produce gene score 3. Gene scores were then categorized by quintiles and association with LVH was assessed by logistic regression analysis.

## Results

### Baseline characteristics LVH cases versus controls

**Figure 1:**
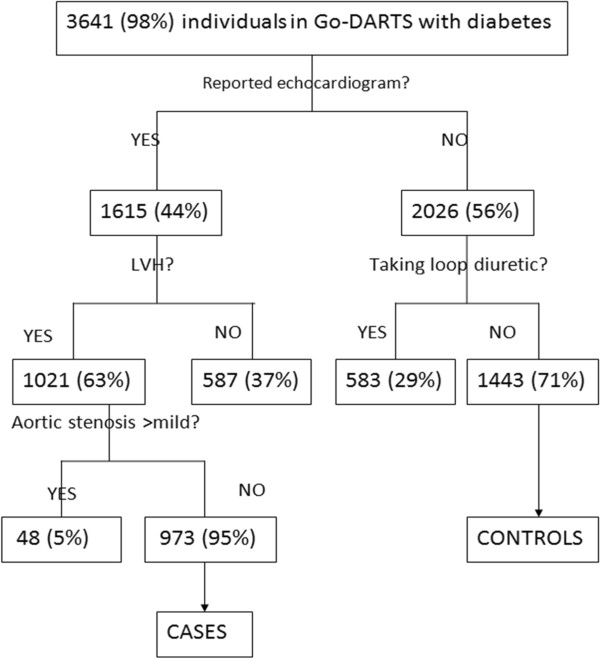
Flow chart summarising inclusion and exclusion cases and controls.

The flow chart in Figure 1 summarises the selection of cases of LVH and controls. As shown, 63% diabetic Go-DARTS participants who had undergone echocardiography had LVH.

Mean baseline characteristics of cases of LVH and controls are shown in Table 1. Logistic regression analysis indicated statistically significant differences between cases of LVH and controls with all variables except gender and systolic blood pressure. Differences in frequency of comorbidities between cases of LVH and controls are also shown in Table 1.

**Table 1:** Comparison of baseline characteristics in LVH cases versus non-LVH controls according to multiple logistic regression analysis

**Variable: mean (SD)**	**Cases (n=973)**	**Controls (n=1443)**	**P value**	**OR**	**95% CI**
Male %	52.62	58.90	0.94	1.01	0.78-1.30
Age years	66.90 (9.83)	62.15 (11.10)	<0.01	1.05	1.04-1.06
SBP mmHg	140.76 (23.07)	139.85 (17.47)	0.07	1.01	1.00-1.01
DBP mmHg	75.09 (13.48)	78.19 (9.97)	<0.01	0.98	0.97-0.99
Height m	1.66 (0.09)	1.68 (0.10)	<0.01	0.04	0.01-0.15
Weight kg	85.85 (18.76)	84.00 (17.10)	<0.01	1.03	1.02-1.03
HbA1C %	7.70 (1.11)	7.63 (1.08)	<0.01	1.2	1.11-1.31

### Survival analysis: outcomes in LVH cases versus controls

**Figure 2:**
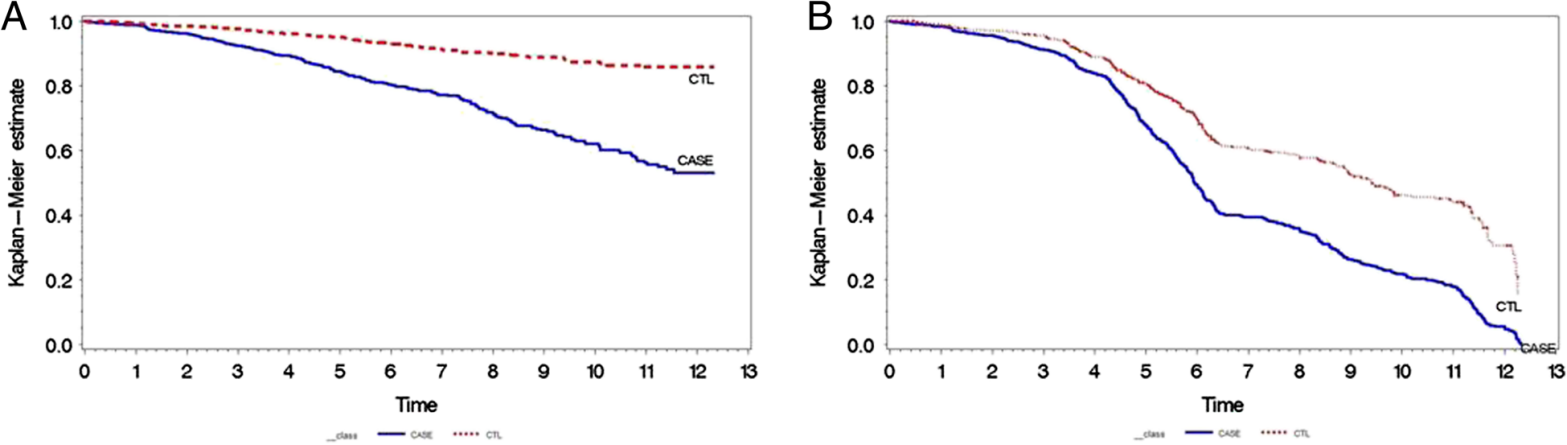
**Variation in survival time in cases of LVH versus non-LVH controls. A **– Kaplan-Meier plot illustrating difference in years to death in LVH cases versus non-LVH controls. **B **- Kaplan-Meier plot illustrating difference in years to death or hospitalization due to cardiovascular disease in LVH cases versus non-LVH controls.

There were 356 (15%) deaths during the median follow up period of 5.62 years (SD 2.64). Mortality in cases of LVH exceeded that of controls as shown in Figure 2A. There was a statistically significant difference in mortality comparing controls and cases (p<0.01, hazard ratio 2.44, 95% CI 1.93-3.09). There was also significantly increased risk in LVH cases for composite outcome of death or hospitalization due to cardiovascular disease according to proportional hazard regression analysis (p<0.01, hazard ratio 2.18, 95% CI 1.89-2.52) as shown Figure 2B.

### Logistic regression analysis and Co-variates

When testing the association with LVH, gender and systolic blood pressure were not statistically significant while diastolic blood, height and age were significant. These results are summarised in Table 1.

### Previously characterised SNPs associated with LVH

Six out of nine SNPs previously associated with LVH through GWAS had beta values showing the same directionality in our study. Rs17132261 showed a significant association with case–control status and had a minor allele frequency (MAF) of 0.01 in our population. Our results showed every copy of the ‘T’ allele was associated with increased risk of LVH (Table 2, p=0.02, beta 0.73, SE 0.31, OR 2.03, 95% CI 1.10-3.73). Rs2292462 showed a significant association with LVH and had a MAF of 0.45 in our population. Every copy of the ‘G’ allele was associated with decreased risk of LVH (Table 2, p=2.26 × 10^-3^, beta -0.20, SE 0.06, OR 0.82, 95% CI 0.73-0.93).

**Table 2:** Comparison between previously published SNP results and results obtained in this study

**SNP**	**Initially published by**	**EA**	**EAF**	**Hardy-Weinberg equilibrium**	**Published beta**	**Published P value**	**Study beta**	**Study SE**	**Study p values**	**Meta-p value**	**Effect on outcome P value (SE)**	**Effect on outcome hazard ratio**	**Effect on outcome 95% CIs for HR**
rs17568359	Vasan et al.	C	0.06	0.35	-4.78	8.53x10^-8^	-0.16	0.13	0.23	1.75x10^-6^	0.29 (0.10)	0.90	0.73-1.10
rs7565161	Vasan et al.	A	0.44	0.75	-3.01	3.19x10^-7^	0.09	0.07	0.19	5.35x10^-6^	0.69 (0.06)	0.98	0.87-1.09
rs7910620	Vasan et al.	G	0.01	1.00	0.17	5.62x10^-9^	-0.44	0.42	0.29	1.45x10^-8^	0.74 (0.58)	0.83	0.27-2.61
rs2059238	Vasan et al.	A	0.23	0.31	-0.02	1.89x10^-7^	-0.06	0.07	0.45	5.07x10^-7^	0.56 (0.05)	1.03	0.93-1.15
rs17132261	Vasan et al.	T	0.01	1.00	0.06	3.36x10^-7^	0.73	0.31	0.02	1.03x10^-8^	0.54 (0.20)	1.13	0.76-1.68
rs6797133	Shah et al.	A	0.39	0.66	-3.7	1.2x10^-7^	3.73x10^-3^	0.06	0.95	2.42x10^-7^	0.74 (0.05)	1.02	0.92-1.12
rs2292462	Shah et al.	G	0.45	0.06	-218.6	3.2x10^-9^	-0.20	0.06	2.26x10^-3^	5.86x10^-10^	<0.01 (0.05)	0.87	0.80-0.96
rs4966014	Shah et al.	C	0.31	0.33	-181.8	1.3x10^-7^	-0.02	0.07	0.74	3.35x10^-8^	0.13 (0.06)	0.92	0.82-1.03
rs2290893	Shah et al.	G	0.64	0.77	-201.4	3.7x10^-8^	-1.57x10^-3^	0.06	0.98	1.70x10^-7^	0.17 (0.05)	0.94	0.85-1.03

*EA *refers to ‘effect allele’, *EAF *to ‘effect allele frequency’, *SE *to ‘standard error’. The outcome referenced in the final 3 columns is a composite outcome referring to time to death or hospital admission due to cardiovascular disease.

Repeating the logistic regression analysis following division of cases into those with increased LV mass and those with concentric/eccentric remodelling showed rs17132261 remained significant (LV mass phenotype p=0.01, increased wall thickness phenotype p=0.01) as did rs2292462 (LV mass phenotype p<0.01, increased wall thickness phenotype p<0.01).

**Figure 3:**
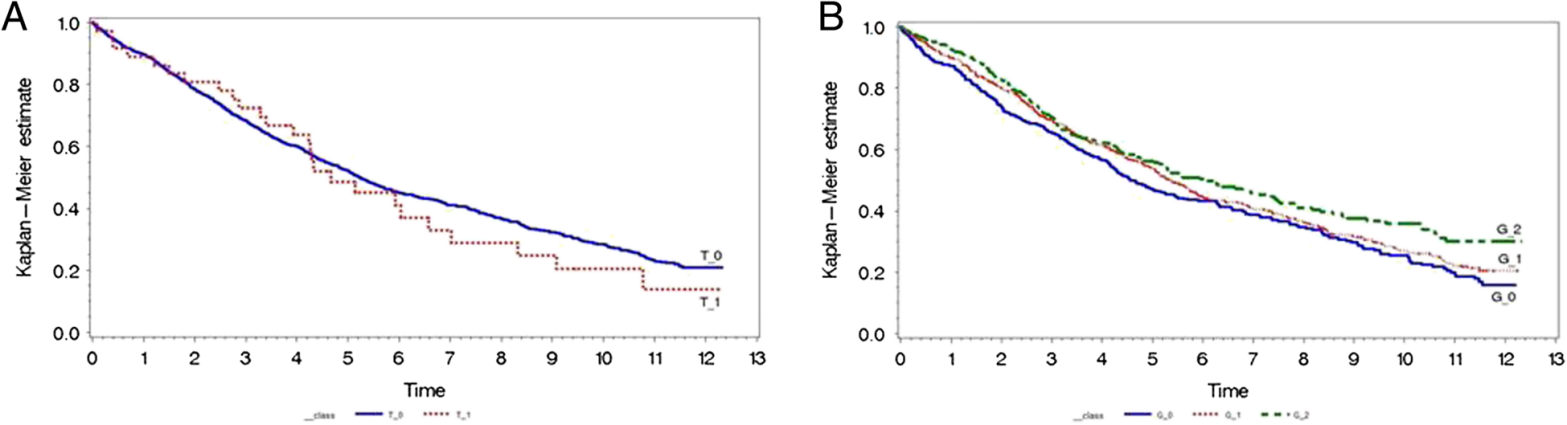
Survival time varies with genetic variants influencing left ventricular hypertrophy.

Rs17132261 variation did not predict mortality and morbidity, (Figure 3A, Table 2) but each copy of the ‘G’ allele variant in rs2292462 showed a clear, dose-dependent relationship with cardiovascular hospitalization and mortality, which was attenuated but remained significant when covariates were accounted for (Figure 3B, Table 2).

### Meta-analysis results

Results from the meta-analyses are displayed in Table 2. A total of 15 028 individuals were analysed for the SNPs published by Vasan *et al.*[20] and 24 449 individuals were analysed for the SNPs published by Shah *et al.*[21]. Investigators within both of these studies sought to identify SNPs associated with LVH, whilst Vasan and colleagues looked at echocardiographically defined LVH, Shah and colleagues looked at electrocardiographically defined LVH. Notably, the combined p-values for rs7910620, rs17132261, rs2292462 and rs4966014 exceeded the threshold for genome wide significance (p<5.0 ×10 ^-8^). Although not significant in our study alone the significance for rs4966014 had been previously published as 8x10^-7^ and achieved genome wide significance (1.35x10^-8^) after the addition of our data. This SNP lies within an intron of the IGF1R gene.

### Gene score calculation and association with LVH

Logistic regression analysis showed gene scoring based on gene score 1 did not predict LVH. However, gene scores 2 and 3 were significant predictors of LVH, as summarised in Table 3.

**Table 3:** Association between gene scoring based on previously published SNPs and LVH

**Gene score**	**OR**	**95% Confidence intervals**	**P value**
Vasan SNPs	1.01	0.94-1.07	0.87
Shah SNPs	1.10	1.03-1.17	4.52x10^-3^
Vasan-Shah SNPs	1.09	1.03-1.16	6.24x10^-3^

## Discussion

### Key findings

To the best of our knowledge, our study represents the first genetics study investigating LVH in T2DM. There were 3 major findings in this study. Firstly, 63% of patients with T2DM undergoing echocardiographic examination had LVH, comparable to previous studies quoting 65% [9]. Secondly, the presence of LVH is independently associated with greater mortality in our cohort compared to controls, which is consistent with findings in previous studies [40]. Thirdly, in this genetics study of a large population of patients with T2DM in Scotland, SNPs previously identified to be predictors of LVH in the general population were replicated.

### The genetic basis of LVH in T2DM

Diabetes remains an independent predictor of LVH. However, not all patients with T2DM develop LVH and those developing LVH do so to varying degrees [11], implying LVH in T2DM has a genetic component. The genetic basis of LVH has been studied in the general population and variants in genes coding for ACE and the beta 1 adrenergic receptor were associated with LVH [41,42]. Variants at these loci were not described in the previous GWAS publications looking at LVH and showed no sign of association in our study. This has been a common feature of the transition from candidate gene studies to GWAS, although notable exceptions include the PPARG Pro12Ala variant for T2DM [43].

### Considerations in investigating the genetic aspect of LVH in T2DM

Our results suggest susceptibility to LVH in T2DM has a detectable genetic component, though investigating any aspect of LVH is intrinsically difficult as there is still discord regarding the optimum way to measure left ventricular size. Electrocardiography is the least expensive method of detecting LVH but the sensitivity is poor in T2DM; echocardiography is more sensitive [9]. Multiple methods of assessing LVH by echocardiography exist including direct 2D measurements, calculation of relative wall thickness and increase in left ventricular mass. Left ventricular mass calculation by echocardiography is the gold standard measure [5,29,30] but complex calculations are involved making direct 2D measurements more popular clinically.

Lack of consensus in defining LVH has also made it difficult to compare genetic findings across studies. How LVH influences outcome is the most important consideration and we have demonstrated our definition strongly predicts mortality. This is compounded by wide variation in the populations from which cases of LVH were extracted prior to genotyping. Initial studies looked at LVH familial correlations [44] whilst later work sought variations in candidate genes in hypertensive patients [17,18] in which LVH was associated with variation in the GNB3 gene encoding the G-protein β_3_ subunit.

Genetic variations frequently influence more than one disease phenotype. A recent study by Povel *et al.* showed SNPs associated with waist circumference (rs17782312 within MC4R) and insulin resistance (rs2943634 within IRS1) were also associated with the metabolic syndrome [45]. Genetic variants associated with features such as serum lipid levels, insulin-resistance and waist circumference in T2DM may also be associated with LVH. Additionally, SNPs may interact with one clinical parameter to influence another. Yin and colleagues found variation in 8 SNPs interacted with obesity to influence serum lipid levels but did not influence serum lipid levels in normal weight individuals [46]. SNPs may interact with other clinical features, such as obesity and lipid levels, to promote LVH in T2DM. This may be an area for future research.

### The role of GWAS

GWAS have broader scope to identify many genetic variants associated with a disease compared to candidate gene studies. However, only two large, population-based GWAS looking at LVH have been performed [20,21]. Vasan *et al.* compared cases of echocardiographically defined LVH with controls across 5 discovery cohorts (n=12 612) and 2 replication cohorts (n=4 094). Multiple SNPs were linked to LVH, but none were replicated [20]. Shah *et al.* genotyped 10 256 individuals from 3 population based cohorts, identifying cases of LVH by ECG criteria and comparing to non-LVH controls. Four SNPs associated with LVH at genome wide significance were also significant in their replication cohort (n=11 777, [21]) but were not significantly associated with LVH in the population based ECHOgen study.

### Physiological relevance of replicated SNPs

Two out of nine previously identified SNPs were replicated at the 1 in 20 level, suggesting true replication. Rs17132261 [20], is found near the SLC25A46 gene that codes for a mitochondrial phosphate transporter [47]. Its minor allele frequency (MAF) in the general population is 0.11 [48]. This proved to be a rare variant within our population (MAF 0.01) and no homozygotes were identified. This was the only SNP that showed any sign of replication for left ventricular thickness in Stage 2 of the study by Vasan *et al.*, and is the only one that we have found to be associated with LVH. Abnormal myocardial energetics may be significant in cardiac pathophysiology [49], so variation here may logically be associated with LVH.

The second replicated SNP, rs2292462, was published by Shah *et al.*[21] and is found in the NMB gene, which has been linked with satiety and weight regulation [50]. Rs2292462 has a MAF of 0.34 in the general population [48], with a MAF of 0.45 in our population. The Kaplan- Meier plot in Figure 3B shows the guanine base variant at rs229462 effects outcome in an allelic additive manner. A SNP in IGF1R (rs4966014) became genome wide significant (3.35 X 10^-8^) in the combined meta-analysis after inclusion of our data, despite this not being significant in our dataset. This was the most robustly associated gene in the study by Shah *et al*. The role of IGF1 signalling in cardiac hypertrophy is well established [51,52] and the SNP lies immediately adjacent to a regulatory region recently identified by the ENCODE as being marked as transcriptional active in muscle cells, by Histone acetylation and DNAse 1 sensitivity [51]. This illustrates that it is unclear which, if any of the other loci were not replicated due to a lack of power, or a true difference in the determinants of echocardiographically defined LVH and ECG-LVH.

Variation in rs2292462 was not predictive of obesity in this study and was associated with LVH when weight was taken into account. This suggests its link with obesity is not driving its association with LVH and adverse outcome in our population.

### Study limitations

Firstly, values used for covariates were taken at regular clinical visits rather than at the time of echocardiography. Values closest to the date the echocardiogram was performed were used for cases of LVH and values closest to genotyping date were used for controls. Arguably, if there had been changes in these covariates between the 2 time points it would have biased our results towards the null.

Secondly, the retrospective nature of our study meant we did not have echocardiographic data for our controls. Our identification of controls was based on the hypothesis patients undergoing echocardiography for any reasons were more likely to have genetically-promoted structural cardiac pathology. Patients on loop diuretics were excluded from the control population as they were deemed more likely to have fluid retention related to undiagnosed LVH with diastolic dysfunction. This method allowed us to identify controls without clinically relevant LVH for comparison. The clear difference in mortality between cases of LVH and controls supports our identification of controls, as does the fact that we replicated SNPs previously associated with LVH.

Thirdly, our sample size was small relative to previous studies. Although only 2 previously published SNPs were replicated, 4 published SNPs retained genome wide significance following meta-analysis with our data. This may indicate we had insufficient power to replicate the other 2 SNPs using our data alone.

## Conclusion

In a GWAS of a large population of patients with T2DM, two SNPs previously identified as predictors of LVH were replicated. It is hoped genetic findings will improve risk stratification in diabetic patients so intensive preventative measures can be taken in those at highest risk. Through identification of genetic loci predicting the development of LVH, it may be possible to identify new molecular drug targets although more research is needed.

## Abbreviations

ACE: Angiotensin converting enzyme; ASE: American society of echocardiographers; BNF: British national formulary; BSA: Body surface area; CHI: Community health index; DARTS: Diabetes audit and research Tayside, Scotland; ECG: Electrocardiogram; GNB3: Guanine nucleotide binding protein; Go-DARTS: Genetics of diabetes audit and research Tayside, Scotland; HIC: Health informatics centre; IGF1R: Insulin-like growth factor 1 receptor; IVS: Interventricular septum; LV: Left ventricle; LVH: Left ventricular hypertrophy; LVIDD: Left ventricular internal diameter in diastole; LVPW: Left ventricular posterior wall; MAF: Mean allele frequency; NMB: Neuromedin B; P value: Probability value; Peroxisome: proliferator-activated receptor gamma; RWT: Relative wall thickness; SLC25A46: Solute carrier family 25 member 46; SMR: Scottish morbidity record; SNP: Single nucleotide polymorphism; T: Thiamine nucleic acid; T2DM: Type 2 diabetes mellitus.

## Competing interests

The authors declare that they have no competing interests.

## Authors’ contributions

HMP designed the study, performed the statistical analysis and co-wrote the manuscript. LAD participated in study design and programming for statistical analysis. NvZ participated in the genomics analysis and helped draft the manuscript. ASF participated in study conception and helped draft the manuscript. DHE designed and collected data for the Tayside echocardiography database. ADM designed and collected data for the Go-DARTS study. ADS participated in study conception and design. CNAP helped design the study, collected the genotyping data and helped draft the manuscript. CCL helped design the study and co-wrote the manuscript. All authors have read and approved the final manuscript.

## Authors’ information

HMP is a Clinical Research Fellow in Cardiology at the University of Dundee. CCL is a Professor of Cardiology at the University of Dundee, CNAP is Chair of Pharmacogenetics and Pharmacogenomics, University of Dundee, ADS is Professor of Clinical Pharmacology, University of Dundee.

Colin NA Palmer and Chim C Lang Joint senior authors.
